# Same Old, Same Old? Heterogeneity in Psychosocial Processes and Well-Being in Minority Aging

**DOI:** 10.1093/geroni/igaf122.432

**Published:** 2025-12-31

**Authors:** Heather Farmer, Jeffrey Stokes, Jennifer Ailshire

## Abstract

There are well-established racial/ethnic disparities in health across the life course, and psychosocial processes (e.g., stress, socioeconomic status) are hypothesized to drive these disparities. Scholarship suggests that unique social contexts, lived experiences, and social identities may contribute to variations in health and well-being within and between racial/ethnic groups, but these associations require more attention. This symposium showcases innovative scholarship that elucidates heterogeneity in psychosocial risk and protective factors linked to health and well-being in minority aging. Dr. Forrester will examine how race and sex influence the association between psychosocial factors (e.g., lifetime trauma, chronic stress, wealth) and accelerated aging, as well as whether psychosocial factors interact with accelerated aging to predict cognitive impairment. Dr. Farmer will use dyadic data from older Black couples to examine whether individual-level and vicarious discrimination are associated with a reduction in purpose in life for individuals and their spouses, as well as whether educational attainment moderates these associations. Dr. Nguyen will explore the influence of religious participation on cognitive trajectories in African Americans and whether this association is conditional on age. Finally, Dr. Stokes will examine racial/ethnic differences in the extent to which neighborhood social context predicts later levels of loneliness and cognitive functioning. Collectively, this work highlights heterogeneity in the psychosocial mechanisms associated with health and well-being in the context of minority aging. Future research, policy, and interventions aimed at reducing longstanding racial/ethnic disparities require theoretically-informed, interdisciplinary approaches that incorporate a focus on social context and lived experiences.

